# Secular trend for increasing birthweight in offspring of pregnant women with type 1 diabetes: is improved placentation the reason?

**DOI:** 10.1007/s00125-022-05820-4

**Published:** 2022-10-26

**Authors:** Gernot Desoye, Lene Ringholm, Peter Damm, Elisabeth R. Mathiesen, Mireille N. M. van Poppel

**Affiliations:** 1 Department of Obstetrics and Gynaecology, Medical University of Graz, Graz, Austria; 2 Center for Pregnant Women with Diabetes, Rigshospitalet, Copenhagen, Denmark; 3 Department of Endocrinology and Metabolism, Rigshospitalet, Copenhagen, Denmark; 4 Department of Obstetrics, Rigshospitalet, Copenhagen, Denmark; 5 Department of Clinical Medicine, University of Copenhagen, Copenhagen, Denmark; 6 Institute of Human Movement Science, Sport and Health, University of Graz, Graz, Austria

**Keywords:** Continuous glucose monitoring, Fetal growth, Hyperglycaemia, Neonatal adiposity, Obesity, Overgrowth, Periconception, Placenta, Review

## Abstract

Despite enormous progress in managing blood glucose levels, pregnancy in women with type 1 diabetes still carries risks for the growing fetus. While, previously, fetal undergrowth was not uncommon in these women, with improved maternal glycaemic control we now see an increased prevalence of fetal overgrowth. Besides short-term implications, offspring of women with type 1 diabetes are more likely to become obese and to develop diabetes and features of the metabolic syndrome. Here, we argue that the increase in birthweight is paradoxically related to improved glycaemic control in the pre- and periconceptional periods. Good glycaemic control reduces the prevalence of microangiopathy and improves placentation in early pregnancy, which may lead to unimpeded fetal nutrition. Even mild maternal hyperglycaemia may then later result in fetal overnutrition. This notion is supported by circumstantial evidence that lower HbA_1c_ levels as well as increases in markers of placental size and function in early pregnancy are associated with large-for-gestational age neonates. We also emphasise that neonates with normal birthweight can have excessive fat deposition. This may occur when poor placentation leads to initial fetal undergrowth, followed by fetal overnutrition due to maternal hyperglycaemia. Thus, the complex interaction of glucose levels during different periods of pregnancy ultimately determines the risk of adiposity, which can occur in fetuses with both normal and elevated birthweight. Prevention of fetal adiposity calls for revised goal setting to enable pregnant women to maintain blood glucose levels that are closer to normal. This could be supported by continuous glucose monitoring throughout pregnancy and appropriate maternal gestational weight gain. Future research should consider the measurement of adiposity in neonates.

**Graphical abstract:**

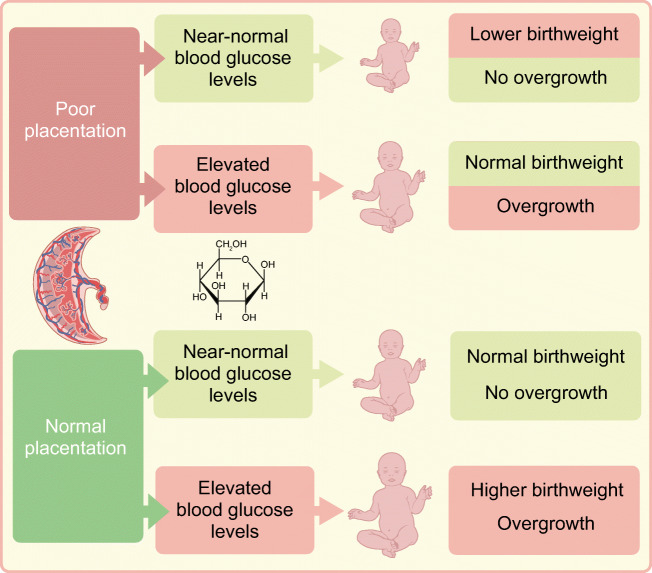

**Supplementary Information:**

The online version contains a slideset of the figures for download available at 10.1007/s00125-022-05820-4.

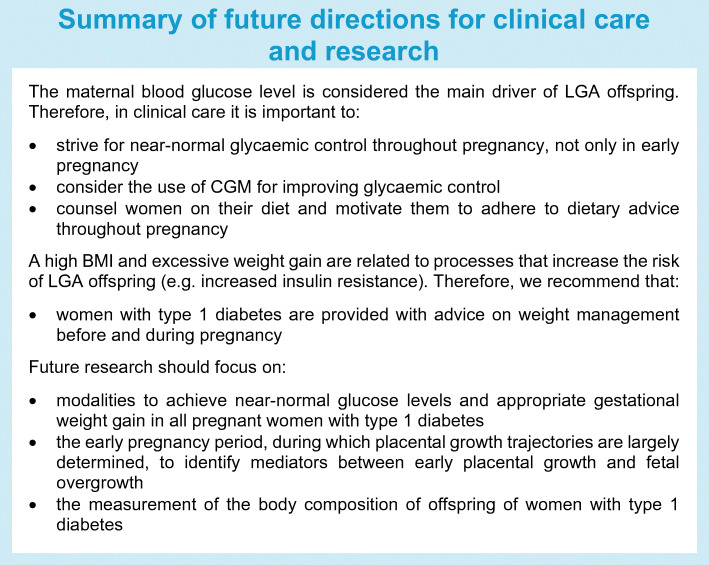



## Introduction

The incidence of type 1 diabetes is rising worldwide, in particular in younger adults [1]. This implies that there is an increasing number of pregnant women with type 1 diabetes. Indeed, in Canada and Scotland, the incidence of pregnancies complicated by diabetes has reportedly increased during the past 15 years [2, 3]. Up to the 1970s, medical attention focused on reducing the rate of congenital malformations and stillbirths. Despite enormous progress in maternal glycaemic control over the past few decades, pregnancies in women with type 1 diabetes still carry manifold maternal and fetal risks. While reduced in frequency, complications in both early pregnancy (e.g. congenital malformations) and late pregnancy (e.g. preeclampsia, preterm labour and stillbirth) are still more prevalent in women with type 1 diabetes than in those without diabetes [4].

In addition, fetal growth is affected in pregnancies with type 1 diabetes. Up to the 1970s the majority of fetuses showed overgrowth, with signs of excessive fat accumulation, but some fetuses grew at a lower rate and showed signs of growth restriction [5]. In more recent decades a paradoxical trend has been observed: neonates born to women with type 1 diabetes increasingly show overgrowth despite improved maternal glycaemic control [6]. A continuous increase in the proportion of large-for-gestational-age (LGA) neonates was found between 1992 and 2013 in Scotland [3] and between 1982 and 2007 in Sweden [7, 8]. The proportion of small-for-gestational-age (SGA) neonates born to women with type 1 diabetes remained stable at around 3–4% up to 2010 in Denmark [9, 10], with a small increase to 6–7% in the last 10 years [11]. In a recent large multicentre trial, rates of LGA offspring were 62–65% and of SGA offspring were 1–2%, depending which growth chart was used [12].

The shift to a higher incidence of fetal overgrowth has short-term implications, such as increased risks of shoulder dystocia, birth trauma and operative delivery [13]. Fetal overgrowth is often based on birthweight and can be defined as a neonate being LGA. However, in diabetes, overgrowth in fetuses actually relates mostly to excessive fat deposition at various anatomical locations [14, 15]. Therefore, fetal overgrowth manifests not only in neonates with increased birthweight, but also in those with normal birthweight [16]. In addition, the concentrations of leptin in the cord blood, presumably derived from fetal adipose tissue, are higher in neonates with overgrowth [17]. In the long-term, offspring of women with type 1 diabetes are more likely to become obese and have increased risks of developing type 2 diabetes, cardiovascular disease and other features of the metabolic syndrome, suggesting a programming role of the adverse intrauterine environment for these health problems [18–20]. Hence, it is important to avoid overgrowth of the fetus throughout pregnancy.

Poor glycaemic control over a longer period may lead to microangiopathy, expressed clinically as diabetic retinopathy and diabetic nephropathy. The presence of diabetic nephropathy in pregnancy is associated with a higher prevalence of SGA infants [9, 21]. Presumably, the presence of microangiopathy is likely to impair placental development and growth, which might be the underlying cause of restricted fetal growth. Reports indicate that the prevalence of nephropathy in pregnant women with type 1 diabetes has decreased in recent decades [9, 11].

In this review, we argue that the increase in birthweight is paradoxically related to the improvement in glycaemic control in the pre- and periconceptional periods over the past few decades. This argument is based on the concept that it is less common for women to experience severe hyperglycaemia and so subsequent processes in early pregnancy around placentation have become less and less affected and microangiopathy lesions of maternal vessels occur less frequently. The improved placentation leads to unimpeded fetal overnutrition as a result of the still present, but milder, maternal hyperglycaemia.

## Determinants of birthweight

Birthweight is determined by the interplay of the genetic makeup of the fetus with macro- and micronutrients supplied by the mother and transferred through the placenta. Thus, the placenta plays an important role throughout pregnancy. In addition to providing nutrients to the fetus, it anchors the fetoplacental unit in maternal uterine tissues, mostly the decidua [22].

**Fig. 1:**
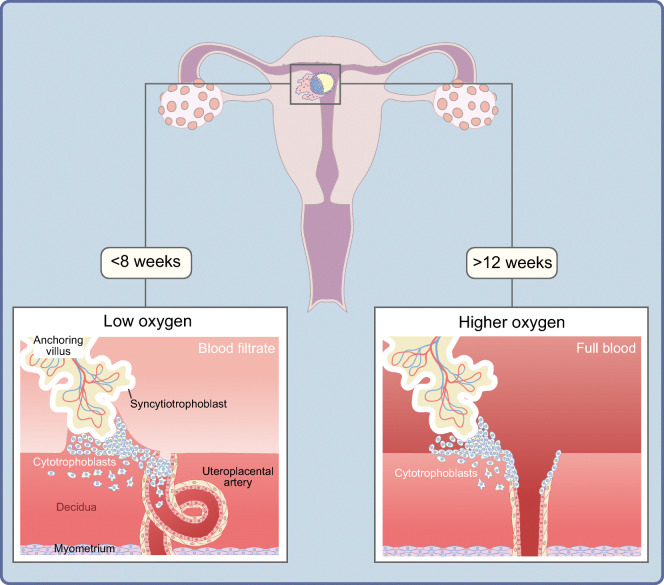
The placenta is made up of a villous tree covered by a continuous cell layer (syncytiotrophoblast). Some villi (anchoring villi) attach to the decidua in the uterus of the pregnant woman (black rectangle in upper part of figure). At the tip of these villi, cytotrophoblasts are amassed. They invade the decidua to physically anchor the placenta in the uterine wall. They also invade uteroplacental (‘spiral’) arteries, where they aggregate to initially form cellular plugs, clogging the vessels. Up to about 8 weeks of pregnancy, only a filtrate of maternal blood with physically dissolved oxygen reaches the intervillous space. Thereafter, the cytotrophoblasts in the spiral arteries contribute to continuously remodel these arteries into high-capacity, low-resistance vessels. Loss of vascular smooth muscle cells leads to widening (opening) of the spiral arteries. As a result, cytotrophoblast plugs are removed, enabling fully oxygenated maternal blood to enter the intervillous space. This is paralleled by a rise in oxygen tension to which the fetoplacental unit is exposed. Each of the spiral arteries contributes to the overall oxygen supply of the fetoplacental unit. Inadequate or absent opening of the 100–150 spiral arteries reduces or inhibits blood flow. Depending on how many arteries are affected, and to what extent, the resulting reduced oxygen supply can ultimately reduce fetal growth. This figure is available as part of a downloadable slideset

In pregnancy, decidual arteries supply the embryo and fetoplacental unit with oxygen and nutrients. In order to accomplish this task, the spiral arteries, that is, vessels that branch from the radial arteries in the myometrium, extending into the decidua, change their lumen to become high-capacity, low-resistance vessels (Fig. 1). This process of spiral artery transformation, or ‘remodelling’ of the spiral arteries, begins early in the first trimester of pregnancy and is critical for adequate oxygen and nutrient supply throughout pregnancy [23, 24].

Up to the 1970s, growth restriction, resulting in SGA neonates, was not an uncommon outcome of pregnancies in women with type 1 diabetes [5]. Chronic fetal hypoxia was often the underlying reason for the growth restriction [25]. Several processes contributed to the increased risk of fetal hypoxia in women with type 1 diabetes.

Type 1 diabetes was often accompanied by diabetic microangiopathy, mostly in individuals with longstanding and poorly controlled type 1 diabetes. When women became pregnant, these vasculopathies affected the uterine arteries, impairing remodelling of the uterine spiral arteries and, thus, reducing blood flow through these supply vessels [26]. This could cause problems with oxygen delivery to the fetoplacental unit and, in extreme cases, result in chronic fetal hypoxia and SGA neonates. In addition, decidual arteries often showed pathological changes, such as decidual vasculopathy in late pregnancy. Lesions in the decidual arteries, such as intramural fibrosis or signs of atherosis, were not uncommon in normotensive women with type 1 diabetes [27, 28], especially in women with diabetic microangiopathy [29]. Furthermore, atheromas in decidual arteries, with resultant elevated vascular resistance [30, 31], may have compounded the problem of diminished blood flow and oxygen supply.

## The role of the pre- and periconceptional periods

Major advances in diabetes management in women with type 1 diabetes [32, 33] have resulted in pre- and periconceptional mean HbA_1c_ levels decreasing from ≥59 mmol/mol (≥7.5%) in 1996–1999 [9] to 52 mmol/mol (6.9%) in 2012–2016 [33]. In addition, blood pressure control has been intensified over the same period. This may have resulted in reductions in the occurrence of decidual vasculopathy and spiral artery lesions. The prevalence of smoking, a well-known risk factor for SGA neonates, has also decreased in the past few decades in Europe and the USA [34]. This may also have contributed to better placentation in early pregnancy. Thus, through overall improved diabetes management and a reduction in smoking, the process of placentation has become near normal. This facilitates adequate oxygen delivery to the placental intervillous space, which reduces the risk of neonates being born growth restricted or SGA.

**Fig. 2:**
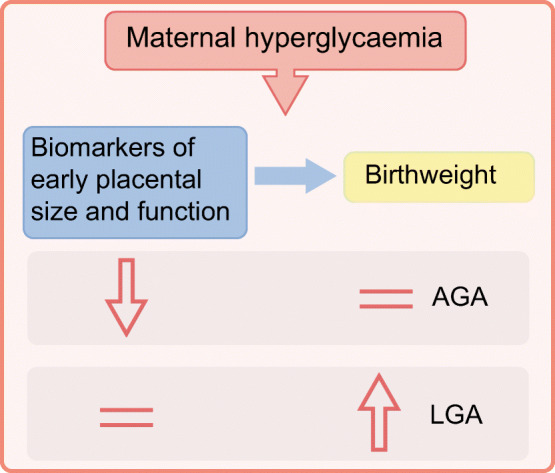
The effect of hyperglycaemia on birthweight category depends on early placental size and function, which can be monitored by biomarkers. An improvement in placentation and early placental function that interacts with hyperglycaemia throughout pregnancy can lead to increased birthweight of neonates born to women with type 1 diabetes. =, unaltered, in the normal range; AGA, appropriate for gestational age. This figure is available as part of a downloadable slideset

Measurements of first trimester concentrations of proteins related to placental size and function support the notion of improved placentation leading to LGA neonates in women with type 1 diabetes [35] (Fig. 2). Proteins used as markers of placentation include placental growth factor, A disintegrin and metalloproteinase 12, placental protein 13, placental growth hormone and pregnancy-associated plasma protein A. Their concentrations in the first trimester of pregnancy in women with diabetes were lower than in women without diabetes, suggesting poor placentation in diabetes. However, combined with the effect of hyperglycaemia, the neonates were born with appropriate for gestational age (AGA) birthweights. Women with diabetes, who had concentrations similar to those in women without diabetes, suggesting that they had ‘healthy’ placentas, gave birth to LGA neonates, again reflecting the contributing effect of hyperglycaemia [35]. Thus, improved placentation and placental function may be associated with a shift in birthweight distribution to higher birthweights and a reduced incidence of SGA neonates born to women with type 1 diabetes.

## Why are more LGA babies born currently?

**Fig. 3:**
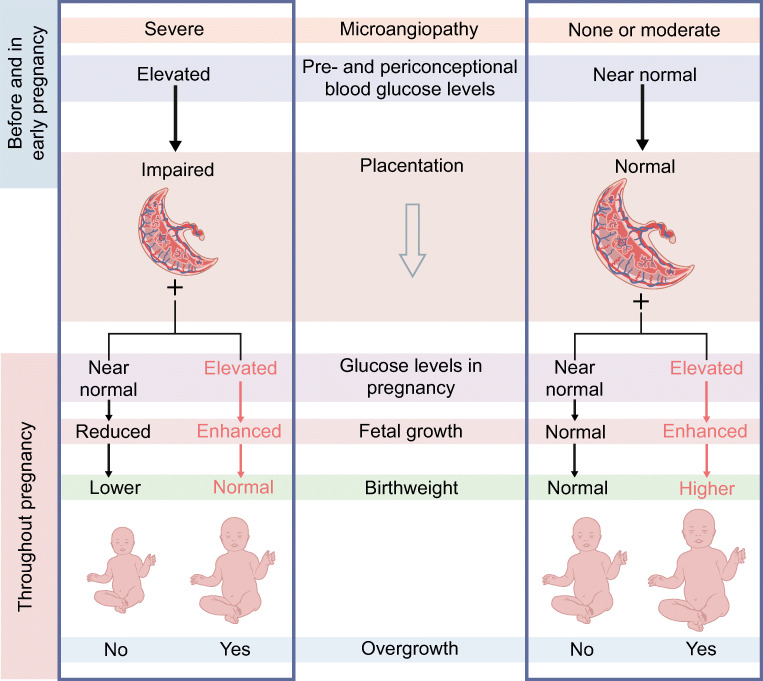
Various scenarios during pregnancy in women with type 1 diabetes showing how the interplay between early placentation and fetal overnutrition in the wake of elevated concentrations of maternal macronutrients can affect birthweight. Only extreme situations are depicted; more graded modifications are possible. The timing of fetal overnutrition in pregnancy (mostly glucose), as well as the genetic background of the fetus, may also play a role. The left side of the figure shows that long-term elevated blood glucose levels in the pre- and periconceptional periods, entailing microangiopathy, and at the time of placentation in early pregnancy may lead to impaired remodelling of spiral arteries, reduced placental blood flow and oxygen transfer. This poor placentation results in the birth of SGA neonates. If this is counteracted by maternal factors such as elevated glucose levels it may result in overnutrition of the fetus through the placenta, leading to fetal overgrowth. This will remain unrecognised if birthweight is the only outcome measure, as these neonates are born with a normal weight. The right side of the figure shows that, with near-normal blood glucose glycaemic levels in the pre- and periconceptional periods, placentation in early pregnancy will be almost normal, that is, with unimpaired or slightly impaired placental blood flow and oxygenation. In this situation, maternal factors such as normal glucose levels may result in normal fetal growth, leading to the birth of AGA neonates, whereas elevated glucose levels may result in overnutrition of the fetus through the placenta, leading to fetal overgrowth. This can manifest in the birth of LGA neonates. Thus, overnutrition can result in two phenotypes of overgrowth independent of birthweight (size of babies): in the presence of microangiopathy or poor placentation, birthweight will be low or normal; in their absence, birthweight will be normal or neonates will be LGA. In both instances, elevated maternal glucose concentrations lead to fetal hyperinsulinaemia and excessive fat deposition as characteristic features of overgrowth in these pregnancies. This figure is available as part of a downloadable slideset

Early pregnancy events that may impair placentation and fetal oxygenation are not the only determinants of birthweight in pregnancies of women with type 1 diabetes. Despite improvements in glycaemic control in type 1 diabetes, maternal and fetal glucose levels are still elevated in both early and late pregnancy [36, 37] and not fully normalised to reflect the physiology of a healthy pregnancy. This is clearly seen in the elevated levels of insulin and C-peptide in cord blood in pregnancies of women with type 1 diabetes [38, 39]. Fetal hyperinsulinaemia stimulates fetal growth, which may result in LGA neonates (Fig. 3).

However, fetal overnutrition may occur even in the case of AGA neonates. In cases of poor placentation with initially reduced fetal growth, hyperglycaemia later in pregnancy may result in neonates being born with an inconspicuous birthweight, that is, being AGA, which then also represents a phenotype of overgrowth with excessive fat deposition. This resembles the thin–fat phenotype in Asian populations, in which neonates are born with a ‘normal’ birthweight but with a disproportionately high body fat percentage compared with neonates from a white population [40] (Fig. 3). If placentation had been adequate, then later hyperglycaemia—even subtle—may have led to an LGA neonate. This distinction between overgrowth in AGA and LGA birthweight categories is important, because it helps explain the results seen for early placental markers and birthweight category [35].

The near-normalisation of decidual vascular health and placentation through improved glycaemic control in the pre-and periconceptional periods may have unmasked the potential for overgrowth in the wake of a still not fully normalised maternal metabolism. Hence, the effects of fetal ‘overnutrition’ as a result of elevated concentrations of maternal macronutrients, particularly glucose, become prominent, leading to more LGA neonates than in previous decades.

In the CONCEPTT trial, a large multicentre RCT of continuous glucose monitoring (CGM) in women with type 1 diabetes [37], women had a mean HbA_1c_ level of 52 mmol/mol (6.9%) at baseline (around 10–11 gestational weeks). The LGA rate was 53% in women randomised to CGM and 69% in those randomised to routine capillary glucose monitoring. These LGA rates were higher than the LGA rate reported in the National Pregnancy in Diabetes UK audit, which was 46%, but early mean HbA_1c_ was higher (60 mmol/mol [7.6%]) than in the CONCEPTT trial [41]. While these studies cannot be directly compared, they provide support for the relevance of early pregnancy blood glucose levels in determining birthweight. However, elevated blood glucose levels in the pre-and periconceptional period are not the only key factor. Rather, early blood glucose levels interact with the influence of glucose levels later in pregnancy to ultimately determine birthweight. The early and late pregnancy periods cannot be separated by a specific date in pregnancy, because the transition varies between pregnancies, depending on a variety of individual factors. Moreover, metabolically induced hyperinsulinaemia in the fetus is an important driver for increasing fetal fat deposition and, hence, contributes to increased birthweight and the overgrowth phenotype [42, 43]. Its onset may vary between individuals and can happen as early as the 14th gestational week [44].

The advance in knowledge over the last decade has enabled us to support earlier concepts about the complex interaction of early and later blood glucose levels and their effects on birthweight, biologically mediated by the quality of placentation in early pregnancy [35, 45]. The role of placental development is highlighted by analyses of a subcohort of the CONCEPTT trial that found higher birthweights in women with type 1 diabetes and suboptimal glucose control when placentas were ‘healthy’, as determined by placental growth factor, than when placentas were ‘unhealthy’ [46].

Insulin may play a contributing role, as seen in the association between the first trimester insulin dose in women with type 1 diabetes and birthweight, which is independent of glycaemic control. The higher the daily insulin dose needed to control glucose levels in the first trimester, the higher the birthweight [47]. Insulin doses in the second or third trimester were not associated with birthweight [47]. One hypothesis is that there is maternal insulin stimulation of early placental growth [48], which is associated with LGA offspring [49]. It remains to be studied whether these LGA neonates have more lean mass and fat mass or whether they represent the classical phenotype of overgrowth with increased fat mass but not lean mass accumulation.

The obesity pandemic has led to more women with type 1 diabetes entering their pregnancy with a pre-pregnancy BMI in the overweight or obese range [50, 51]. This can further exacerbate the risk of having an LGA infant [52]. The specific pathophysiological underpinnings are unclear but may involve maternal hyperglycaemia, because of obesity-associated insulin resistance, with subsequent early fetal hyperglycaemia and hyperinsulinaemia pulling more glucose to the fetus, the so-called glucose steal phenomenon [42]. Although the fetal glucose steal phenomenon has not yet been demonstrated in pregnancies with type 1 diabetes, it is mainly driven by fetal hyperinsulinaemia, which is also present in these pregnancies [38, 39]. However, multiple other pathways affecting birthweight have been proposed [53]. Although the daily insulin dose per unit body weight has not changed profoundly over the last few decades, the increase in body weight of pregnant women may have led to increases in absolute insulin doses, which in turn may have stimulated early placental growth with potential sequelae (see above).

## Why should we avoid fetal overgrowth?

Fetal overgrowth has the potential for short-term complications [4], and the metabolic derangements, leading to excessive fat deposition, pose long-term risks to the offspring [43]. In more than 4800 mother–offspring pairs in the global Hyperglycaemia and Adverse Pregnancy Outcome (HAPO) study, neonatal fat was positively associated with obesity in offspring at age 11 years [54]. Neonatal adiposity was the mediator between the altered intrauterine metabolic milieu and childhood obesity. This finding is important because individuals with a higher number of adipocytes at 2 years and older will have more adipocytes throughout their lifespan [55]. The higher number of ‘hungry’ adipocytes, destined to be filled with triacylglycerols, will then facilitate excessive fat deposition, thereby contributing to the long-term obesity risk [56]. Thus, fetal overgrowth with excessive fat accumulation in pregnancies with type 1 diabetes may lead to similar long-term consequences as in offspring born to women with gestational diabetes or type 2 diabetes or with obesity [18–20].

## What can we do to prevent fetal overgrowth?

Achieving strict glycaemic control throughout pregnancy is mandatory to prevent inappropriate fetal growth. The effect of the in utero environment on fetal growth may be lessened by implementing a diabetes management plan to achieve glucose levels closer to physiological levels and also by considering daily fluctuations of glucose. However, the actual level of glucose needed to maintain growth without increased adiposity is currently unknown. CGM may improve glycaemic control by dampening fluctuations of glucose concentrations, thereby reducing the proportion of LGA neonates [37]. CGM seems to be more effective than the use of insulin pumps at achieving good glycaemic control, which surprisingly has not resulted in fewer LGA infants than multiple daily injections [57, 58]. However, CGM is a costly intervention and may not be available in all resource settings. Diet plans and weight management are less costly alternatives [4].

Maternal pre-pregnancy BMI and gestational weight gain play a role in fetal growth. In normal pregnancy [52], as well as in pregnancy in women with type 1 diabetes [36], excessive gestational weight gain is associated with higher fetal growth, determined by birthweight SD score, which is independent of glycaemic control. Pre-pregnancy and antenatal caregivers could advise women with overweight and obesity on weight management before and during pregnancy. Counselling interventions to improve adherence to healthy eating may be effective in reducing fetal overgrowth in pregnant women with type 2 diabetes [59], but their effect in pregnant women with type 1 diabetes needs to be elucidated in future studies.

Over the last few decades, people’s lifestyles have generally become more obesogenic, including frequent snacking and fewer home-cooked meals. This became even more evident during the lockdown periods of the COVID-19 pandemic [60]. Excessive carbohydrate intake is related to poorer glycaemic control in pregnant women with type 1 diabetes [61] and is associated with adverse pregnancy outcomes. These problems are often related to low socioeconomic status [62]. In addition, the strict glycaemic control regimen and frequent use of CGM and insulin pumps may make pregnant women with type 1 diabetes feel safe and tempt them to eat more [61]. Despite the therapeutic and medical improvements, often resulting in near-normalised blood glucose levels, efforts to motivate women to strictly adhere to their diet plan throughout pregnancy is still of utmost importance [4]. The recommended diet plan should include a sufficient intake of macro- and micronutrients with a minimum daily intake of 175 g carbohydrates with a low glycaemic index [63] and limit gestational weight gain [61].

## Future directions

### Clinical directions

Currently, women with type 1 diabetes have been shown to have fairly good glycaemic control in the periconceptional period, as determined by their HbA_1c_ levels [64]. HbA_1c_ is an appropriate measure of average blood glucose levels, but its suitability as a pregnancy outcome predictor in type 1 diabetes has been questioned [65]. Recently, evidence has been accumulating about the importance of monitoring temporal glucose profiles throughout pregnancy and of glucose levels being in the desired glycaemic range for as much time as possible to improve birth outcomes [66, 67]. This calls for revised goal setting to achieve glucose levels in the physiological range throughout pregnancy (see text box: Summary of future directions). Although CGM holds promise for achieving glucose levels close to physiological levels over 24 h periods during pregnancy [68], euglycaemia may still be difficult to achieve in pregnant women with type 1 diabetes [50]. It is hoped that more and more healthcare systems globally will financially support the use of CGM, similar to the recent approval of these technologies by the UK government.

Lowering BMI before pregnancy and limiting gestational weight gain during pregnancy are mandatory for women with overweight or obesity with type 1 diabetes. However, both reducing pre-pregnancy BMI and preventing excessive gestational weight gain may be challenging and difficult to achieve, despite lifestyle counselling.

### Research directions

The early growth period of the fetus and how it is influenced by the maternal environment is an underexplored area. Recently established, well-phenotyped early pregnancy cohorts [69] will help to gain insight into factors that modify early embryonic and fetal growth in a variety of maternal settings. It is well known that the maternal diet in the pre- and periconceptional periods can influence the early growth of the embryo, fetus and placenta [70], but the effect of metabolic changes in the mother on the embryo and fetus is less well studied. Some influences of maternal type 1 diabetes on early placental development and function have been found [22, 71–73], but their consequences for fetal growth remain elusive. Despite feasibility issues, we suggest that future research should focus on the early pregnancy period during which placental growth trajectories such as size/volume and vascularisation are largely determined [74, 75]. Moreover, fetal hyperinsulinaemia, the key driver of fetal growth and fat accumulation in pregnancies complicated by diabetes, has its roots in early pregnancy [42]. Thus, the early pregnancy period has a strong influence on later fetal growth.

To date, human studies, including intervention studies, have focused on the proportion of LGA neonates born to women with type 1 diabetes. However, neonates with an inconspicuous birthweight can also show overgrowth as a result of their overnutrition in utero against the background of impaired placentation. These offspring constitute an important group for future obesity prevention efforts and should be of major public health concern. The birthweight-centric classification system of AGA/LGA does not capture excessive adiposity, but no alternative classification system is currently available. In the future, it will be important to expand study outcomes to include the number of fetuses with overgrowth with increased adiposity despite having a normal birthweight (see text box: Summary of future directions). This will require measurement of neonatal body composition, although this might be difficult to implement in routine clinical practice. While sophisticated techniques such as air displacement plethysmography (e.g. PEA POD, COSMED) or dual-energy x-ray absorptiometry may be preferred for measurement of body composition, measurement of skinfold thickness using callipers or other techniques can give adequate results that can be converted into body fat and have been widely used in large-scale studies [76]. Furthermore, studies should include measurement of a proxy indicator for placentation (biomarkers, uterine artery blood flow) in the early pregnancy period combined with quantification of blood glucose levels in the pregnant women throughout pregnancy. These exposures could then be related to the outcome of neonatal adiposity.

## Summary and conclusion

The increase in birthweight over time reported in several populations of pregnant women with type 1 diabetes, despite improved glycaemic control, has been of concern. It has prompted the suggestion that, in addition to hyperglycaemia, other factors may account for this observation [51].

Our heuristic concept presented here provides an alternative explanation. We focus on improved placentation in early pregnancy in women with type 1 diabetes as the basis on which the growth-promoting effects of maternal hyperglycaemia later in pregnancy are superimposed. This concept posits that complex interactions of blood glucose levels at different stages of pregnancy ultimately determine birthweight or, more specifically, fetal overgrowth in pregnancies complicated by type 1 diabetes. This calls for more intensive efforts to bring glucose levels closer to the physiological range throughout pregnancy, and the use of modern diabetes technologies may help women to achieve this. Future research should focus on the placentation phase and onwards and include some form of neonatal adiposity measurement in addition to a birthweight-centred growth outcome.

## Supplementary information

Slideset of figures (PPTX 386 KB)

## Abbreviations

AGA: Appropriate for gestational age
CGM: Continuous glucose monitoring
CONCEPTT: Continuous Glucose Monitoring in Women with Type 1 Diabetes in Pregnancy Trial
LGA: Large for gestational age
SGA: Small for gestational age
