# Supplementary material for: Secular trend for increasing birthweight in offspring of pregnant women with type 1 diabetes: is improved placentation the reason?
Source: Diabetologia. 2022 Oct 26;66(1):33–43. doi: 10.1007/s00125-022-05820-4 (PMC9607824; doi:10.1007/s00125-022-05820-4)
Supplement: Supplementary file 1 — (PPTX 386 KB) [file 125_2022_5820_MOESM1_ESM.pptx]

## Slide 1
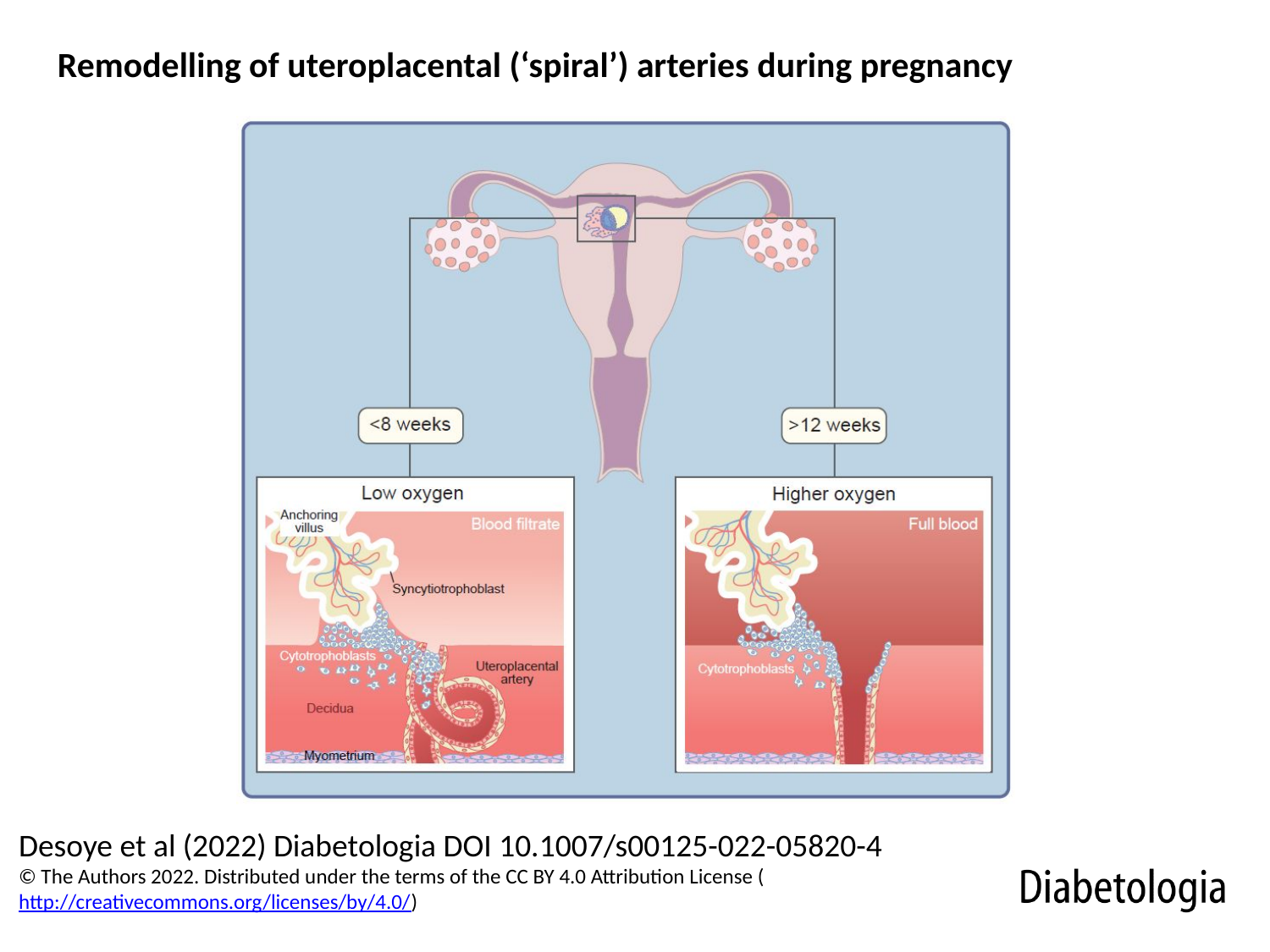

Remodelling of uteroplacental (‘spiral’) arteries during pregnancy
Desoye et al (2022) Diabetologia DOI 10.1007/s00125-022-05820-4
© The Authors 2022. Distributed under the terms of the CC BY 4.0 Attribution License (http://creativecommons.org/licenses/by/4.0/)

## Slide 2
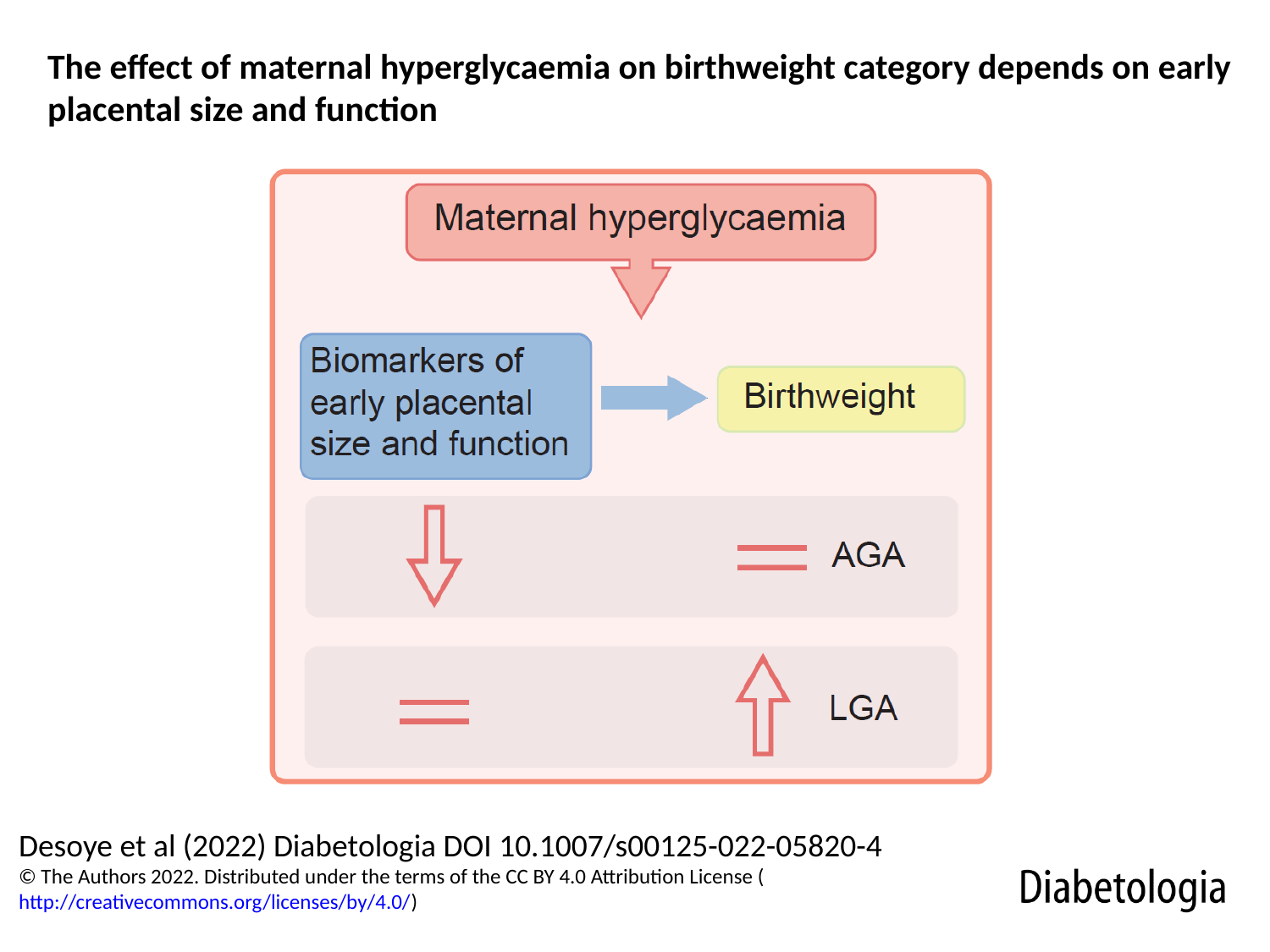

The effect of maternal hyperglycaemia on birthweight category depends on early placental size and function
Desoye et al (2022) Diabetologia DOI 10.1007/s00125-022-05820-4
© The Authors 2022. Distributed under the terms of the CC BY 4.0 Attribution License (http://creativecommons.org/licenses/by/4.0/)

## Slide 3
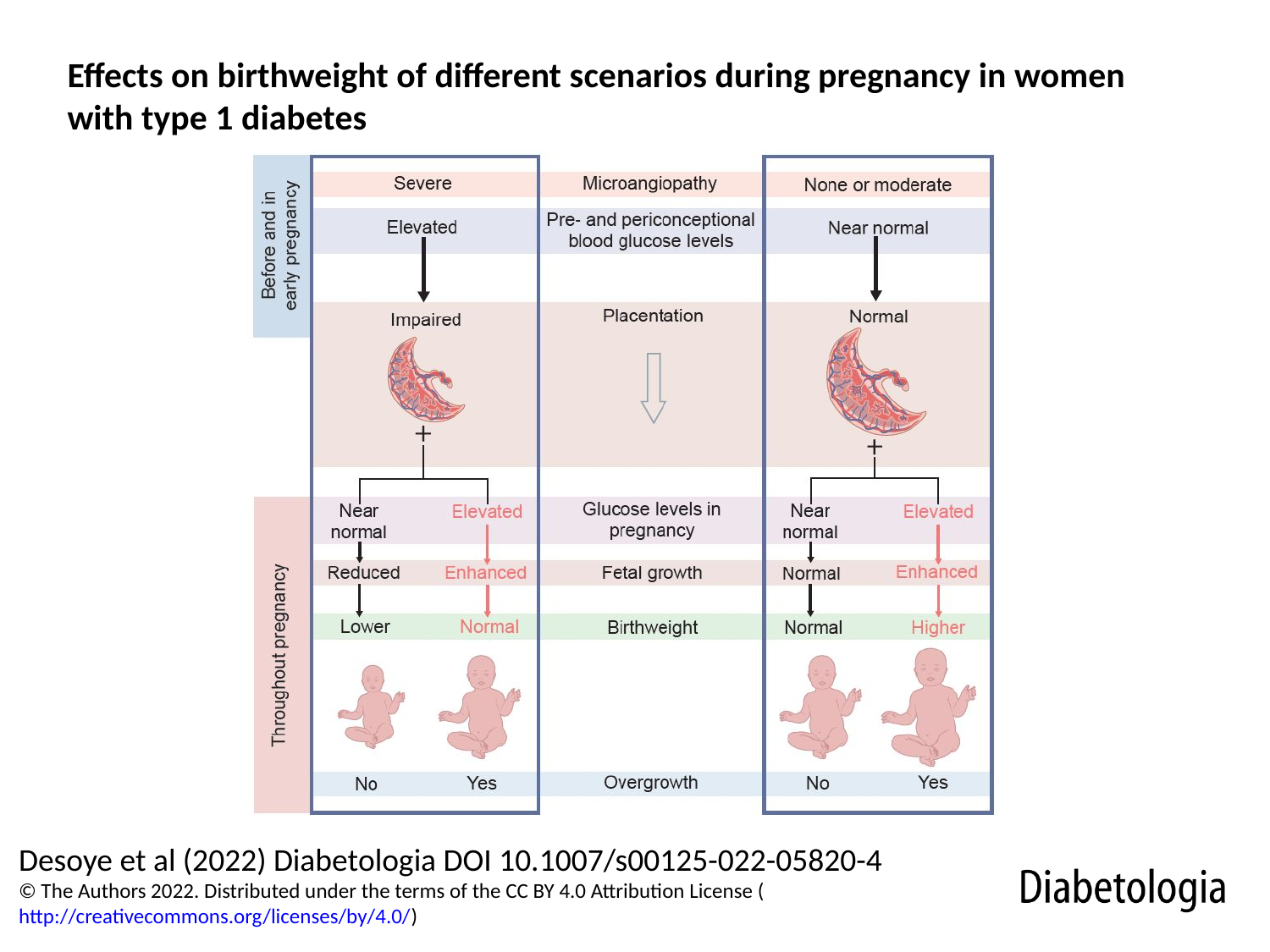

Effects on birthweight of different scenarios during pregnancy in women with type 1 diabetes
Desoye et al (2022) Diabetologia DOI 10.1007/s00125-022-05820-4
© The Authors 2022. Distributed under the terms of the CC BY 4.0 Attribution License (http://creativecommons.org/licenses/by/4.0/)
